# Global Kalman filter approaches to estimate absolute angles of lower limb segments

**DOI:** 10.1186/s12938-017-0346-7

**Published:** 2017-05-16

**Authors:** Samuel L. Nogueira, Stefan Lambrecht, Roberto S. Inoue, Magdo Bortole, Arlindo N. Montagnoli, Juan C. Moreno, Eduardo Rocon, Marco H. Terra, Adriano A. G. Siqueira, Jose L. Pons

**Affiliations:** 1 Department of Electrical Engineering, Federal University of São Carlos, São Carlos, Brazil; 2 Division PMA, Department of Mechanical Engineering, Katholieke Universiteit Leuven, Leuven, Belgium; 3 Neural Rehabilitation Group, Consejo Superior de Investigaciones Científicas, Madrid, Spain; 4 Department of Biomedical Kinesiology, Katholieke Universiteit Leuven, Leuven, Belgium; 5 Group of Neural and Cognitive Engineering of the Consejo Superior de Investigaciones Científicas, Madrid, Spain; 6 Department of Electrical Engineering of the University of São Paulo, São Carlos, Brazil; 7 Department of Mechanical Engineering of the University of São Paulo, São Carlos, Brazil

**Keywords:** Exoskeleton, Inertial sensors, Kalman filter, Markovian jump systems, Wearable robots

## Abstract

**Background:**

In this paper we propose the use of global Kalman filters (KFs) to estimate absolute angles of lower limb segments. Standard approaches adopt KFs to improve the performance of inertial sensors based on individual link configurations. In consequence, for a multi-body system like a lower limb exoskeleton, the inertial measurements of one link (e.g., the shank) are not taken into account in other link angle estimations (e.g., foot). Global KF approaches, on the other hand, correlate the collective contribution of all signals from lower limb segments observed in the state-space model through the filtering process. We present a novel global KF (matricial global KF) relying only on inertial sensor data, and validate both this KF and a previously presented global KF (Markov Jump Linear Systems, MJLS-based KF), which fuses data from inertial sensors and encoders from an exoskeleton. We furthermore compare both methods to the commonly used local KF.

**Results:**

The results indicate that the global KFs performed significantly better than the local KF, with an average root mean square error (RMSE) of respectively 0.942° for the MJLS-based KF, 1.167° for the matrical global KF, and 1.202° for the local KFs. Including the data from the exoskeleton encoders also resulted in a significant increase in performance.

**Conclusion:**

The results indicate that the current practice of using KFs based on local models is suboptimal. Both the presented KF based on inertial sensor data, as well our previously presented global approach fusing inertial sensor data with data from exoskeleton encoders, were superior to local KFs. We therefore recommend to use global KFs for gait analysis and exoskeleton control.

**Electronic supplementary material:**

The online version of this article (doi:10.1186/s12938-017-0346-7) contains supplementary material, which is available to authorized users.

## Backgound

Most of the current motor rehabilitation interventions are based on highly repetitive and task-oriented exercises [1]. Exoskeletons have been developed to assist in delivering this repetitive and intensive therapy, and thus alleviate the load on physiotherapists. Exoskeletons are wearable robots with soft [2], or rigid structures [3, 4] that are directly attached to the patient.

**Fig. 1:**
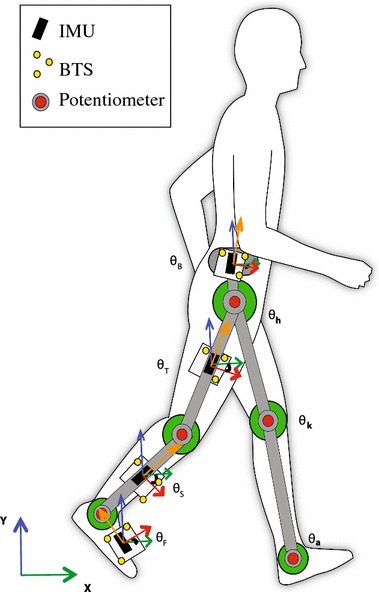
Experimental setup of a healthy person wearing the H2 exoskeleton, with passive BTS-markers (Optoelectronic system with reflexive markers from BTS Bioengineering, Italy) and inertial sensors. The inertial sensors and BTS-markers are attached to the exoskeleton using rigid plates

A large number of exoskeleton control strategies rely on a reference trajectory obtained from healthy subjects. Based on feedback from relative joint angles and/or absolute segment angles during gait, the exoskeleton assists the patient in performing this reference motion, see Fig. 1. In clinical gait analysis, these angles are used to describe and analyze the walking abilities of patients and athletes. Obtaining accurate absolute angles is thus essential for both exoskeleton control and dynamic gait analysis. However, sensors embedded in the exoskeleton, such as encoders and potentiometers, can only provide relative angles. The error between these measured relative angles and the anatomical joint angles from the patient, strongly depends on the design of the exoskeleton and the attachment of the exoskeleton to the patient. In order to obtain the absolute orientation of the exoskeleton or to correct the error in the encoder measurements, additional sensors such as inertial sensors are used [5, 6].

Inertial measurement units (IMUs) commonly consist of three dimensional accelerometers and gyroscopes. IMUs, or magnetic and inertial measurement units (MIMUs), have demonstrated their effectiveness in clinical practice in applications ranging from instrumented clinical testing [7–9] fall detection and analysis [10], to gait segmentation [11] and gait analysis [12].

Absolute orientation estimation (orientation with respect to a global frame) is usually performed by MIMUs [13, 14]. In MIMUs, 3D orientation with respect to the earth frame is computed by integrating the gyroscope signals from a known starting condition, provided by the accelerometers and magnetometers. Noise in the gyroscope signal causes an unbounded error when integrated [15]. Accelerometers and magnetometers are therefore used to correct the orientation estimate based on the integrated gyroscope signals.

In Kalman filtering, two main approaches are used to update the gyroscope-based orientation estimate using the accelerometer data. The first is a modeling approach, where the the acceleration signal is separated into gravitational acceleration and acceleration due to motion [16, 17]. Only the modelled gravitational acceleration is subsequently used to determine the inclination of the sensor in the KF. The second approach is a threshold-based method where the accelerometer data is only used to determine inclination in the KF when static or quasi-static periods are detected [6, 18]. To facilitate comparison, all the methods discussed in this paper use the threshold-based approach.

The most common threshold considers the norm of the measured acceleration [19]. When the norm of the measured accelerations of a specific sensor compared with the gravitational constant ($g=9.81~{\text{ m/s}^2}$) is smaller than an arbitrary bias, the acceleration measurement of that sensor is considered reliable and it is used in the KF. When this comparison exceeds the threshold, the measurements are deemed unreliable and therefore not used to update the gyroscope-based estimation [6, 19]. Using this approach, each sensor’s estimate is only updated with accelerometer data when the criterion is met by that sensor, irrespective of what the other sensors are measuring. However, under more dynamic conditions the number of instances where the accelerometer measurements are deemed reliable can be severely reduced. This can potentially lead to a lack of updates for segments with fewer relative static incidences due to their particular acceleration pattern, as is the case of the body.

The aforementioned methods use local models, meaning that they only use information from each sensor individually to update the estimates. The authors have previously presented a global and cooperative approach to address the limitation of local KF approaches in [6]. In the global models, measurements of all sensors are assumed to be related to each other. The updates of each sensor are thus based on information provided by various sensors. In [6], we presented an approach related to MJLS-based KF, combining four inertial sensors and three encoders from one exoskeleton’s leg. At each time instant, the inertial sensors are evaluated based on the norm of the measured acceleration. If one or several sensors meet the criterion, the sensor that best meets the criterion is used to update the collective. The update is therefore based on the information of the best inertial sensor and three encoders. This cooperative approach, using Markovian jumps when switching between IMUs, led to more frequent updates and better estimates when compared to local methods. However, the algorithm proposed in [6] is only applicable when information from encoders is available, and was only tested on simulated data. In [20] we presented results in order to verify the influence of different types of inertial sensors calibration to local and global approaches.

In this paper we present a novel global KF, the matricial global KF, based uniquely on inertial sensor data. We furthermore validate the proposed KF presented here, as well as the MJLS-based KF presented in [6], using experimental data. We also include a comparison between the commonly used local KF and the aforementioned global KFs to demonstrate the strength and benefits of global KFs over local KFs. We hypothesize that global filters will outperform the local filter models.

## Methods

We first provide a brief introduction to matricial local and MJLS-based models. Subsequently, we introduce the newly developed matricial global model that is based only on inertial sensor data. A more detailed explanation on local and MJLS-based models can be found in Additional file 1 or in [6].

### Local models

When applied to inertial sensor data, a local KF is a filter that only uses information from a specific IMU. In the specific case of gait analysis, the local state-space model to estimate absolute segment orientation based on a sensor attached to the lower limb is formulated as:

1 $$\begin{aligned} \dot{x}&= Ax(t) + Bw(t), \nonumber \\ \left[ \!\begin{array}{r} \Delta \dot{\theta }(t)\\ \Delta \dot{b}_{\text {g}}(t)\end{array}\!\right]&= \left[ \!\begin{array}{rr} 0 &{} 1 \\ 0 &{} - \frac{1}{\tau _{\text {g}}} \end{array}\!\right] \left[ \!\begin{array}{r} \Delta \theta (t)\\ \Delta b_{\text {g}}(t)\end{array}\!\right] + \left[ \!\begin{array}{rr} 1 &{} 0 \\ 0 &{} 1 \end{array}\!\right] \left[ \!\begin{array}{r} \eta _\text {g}(t)\\ \eta _\text {b}(t) \end{array}\!\right] , \end{aligned}$$

where $\Delta \theta (t)= \theta - \hat{\theta }_\text {g}$ and $\Delta b_{\text {g}}(t)$ are respectively the orientation estimate and offset errors of the gyroscope, $\theta$ is the absolute angle and $\hat{\theta }_\text {g}$ is the angle estimate calculated from the gyroscope, $\eta _\text {g}(t)$ and $\eta _\text {b}(t)$ are the Gaussian white noises of the gyroscope and the gyroscope bias, and $\tau _{\text {g}}$ is the Markov process correlation time [6, 18]. Since only the orientation estimate is observable, the output equation is given by:

2 $$\begin{aligned} z = Cx(t) + v(t) = \left[ \begin{array}{rr} 1&0 \end{array}\right] \left[ \begin{array}{r} \Delta \theta (t)\\ \Delta b_{\text {g}}(t)\end{array}\right] + \eta _\text {a}(t), \end{aligned}$$

where $\eta _\text {a}(t)$ is the Gaussian white noise of the accelerometer. In the filtering implementation, we use $z=\hat{\theta }_\text {a}-\hat{\theta }_\text {g}$, where the orientation estimate from the accelerometer ($\hat{\theta }_\text {a}$) is considered a reliable measurement and replaces the absolute angle ($\theta$).

Once the state-space model and the output equation are defined, KF-based algorithms can be used to estimate the state which represents the correction term $\Delta {\hat{\theta }}$. So the absolute angles can be computed as:

3 $$\begin{aligned} \hat{\theta } (t) = \hat{\theta }_{\text {g}} (t)+ \Delta \hat{\theta } (t), \end{aligned}$$

where $\hat{\theta }_{\text {g}}$ is the absolute angle based only on gyroscope data, and $\hat{\Delta } \theta$ the absolute angle estimated by KF.

#### Matricial local model

Consider now the problem of estimating the absolute angles of the lower limbs, specifically the absolute angles of body, thigh, shank, and foot segments. For sake of simplicity, only one leg is considered. We can use one local model for each segment in a matrix arrangement. The state-space and output equations can be summarized as:

4 $$\begin{aligned} \dot{x}(t)&= \bar{A} x(t) + \bar{B} w(t), \end{aligned}$$

5 $$\begin{aligned} z(t)&=\bar{C}(t) x(t) + v(t), \end{aligned}$$

the state vector is defined as $$x = \left[ {\begin{array}{*{20}{c}} {x_B}&{x_T}&{x_S}&{x_F} \end{array}} \right] ^T$$, with $$x_i = \left[ {\begin{array}{*{20}{c}} {\Delta \theta _i}&{\Delta b_{i}} \end{array}} \right]$$; ${\Delta \theta _i} = \theta _i - \hat{\theta }_{i_\text {g}}$, are the errors between the absolute angles ($\theta _i$) and the angle estimates calculated by the gyroscopes ($\hat{\theta }_{i_\text {g}}$); and $\Delta b_{i}$, are the errors of the bias generated by the gyroscopes for each segment, for $i=\{B,T,S,F\}$, where *B*, *T*, *S*, and *F* stand for body/trunk, thigh, shank, and foot segments, respectively. The vectors *w*(*t*) and *v*(*t*) contain the Gaussian white noise of the gyroscopes, gyroscope bias, and accelerometer respectively. The output matrix $\bar{C}$ is defined as:

6 $$\begin{aligned} \bar{C} = \left[ \begin{array}{llllllll} 1&{}0&{}0&{}0&{}0&{}0&{}0&{}0\\ 0&{}0&{}1&{}0&{}0&{}0&{}0&{}0\\ 0&{}0&{}0&{}0&{}1&{}0&{}0&{}0\\ 0&{}0&{}0&{}0&{}0&{}0&{}1&{}0 \end{array}\right] . \end{aligned}$$

In this local approach, named here matricial local model, the state-space and output equations are both diagonal or block-diagonal. The system can thus be considered uncoupled. A complete description of this model and its KF algorithm can be found in Additional file 1.

### Global models

Global models are composed of matrix arrangements by the same state-space (4) of the matricial local model. The difference lies in the choice of the output matrix, and, as consequence, in the update equations of the KF Algorithms.

#### MJLS-based model

The Markovian Jump Linear Systems-based model presented in [6] fuses inertial sensors with relative joint sensors embedded in the exoskeleton. Only the IMU that best meets a given threshold criterion is used. We provide a brief overview of the model below, but refer to [6] and Additional file 1 for a more detailed description.

The MJLS-based model for absolute angular estimation of lower limb exoskeletons can be described by the following state-space equations:

7 $$\begin{aligned} \dot{x}(t) &= \bar{A} x(t) + \bar{B} w(t), \end{aligned}$$

8 $$\begin{aligned} z(t) &= \bar{C}_{\Xi }(t) x(t) + v(t), \end{aligned}$$

where $\Xi (t) \in \{B,T,S,F\}$ defines the possible Markovian jumps. The vector of output measurements is defined as:

9 $$\begin{aligned} z = \left[ \begin{array}{llll} {\Delta _{IMU}}&{\Delta \theta _{h}}&{\Delta \theta _{k}}&{\Delta \theta _{a}} \end{array}\right] ^T, \end{aligned}$$

where $\Delta _{IMU}=[\hat{\theta}_{{\text{a}}_{IMU}}-\hat{\theta}_{{\text {g}}_{IMU}}]$ are the errors between the estimates of the absolute angles calculated for each segment by the accelerometers ($\hat{\theta }_{\text {a}_{IMU}}$) and the estimates calculated by the gyroscopes ($\hat{\theta }_{\text {g}_{IMU}}$); and $\Delta \theta _{j}=[\Delta \theta _{i} - \Delta \theta _{i+1}]$, for $j = \{h,k,a\}$, are the errors of the relative angles of the corresponding joints, *j*, where *h*, *k* and *a* stand for hip, knee, and ankle. The output matrix $\bar{C}_{\Xi }$ is defined as:

10 $$\begin{aligned} \bar{C}_{\Xi }(t) \!=\! \left[ \begin{array}{llllllll} M_{B}(t) &{} 0 &{} M_{T}(t) &{} 0 &{} M_{S}(t) &{} 0 &{} M_{F}(t) &{} 0\\ 1 &{} 0 &{} { - 1} &{} 0 &{} 0 &{} 0 &{} 0 &{} 0\\ 0 &{} 0 &{} 1 &{} 0 &{} { - 1 } &{} 0 &{} 0 &{} 0\\ 0 &{} 0 &{} 0 &{} 0 &{} 1 &{} 0 &{} { - 1} &{} 0 \end{array}\right] , \end{aligned}$$

where $M_{i}$, for $i=\{B,T,S,F\}$, assumes values of zero or one, according to the angle associated with the value of $\Delta _{IMU}$.

The criteria for reliable accelerometer measurements and the Markovian state in discrete time are defined as:

11 $$\begin{aligned} \rho (k)& := \mathop {\min }\limits _i \left( \left| \Vert a_i\Vert - g \right| \right) , \end{aligned}$$

12 $$\begin{aligned} \Xi (k)& := \mathop {\arg } \mathop {\min }\limits _i \left(\left| \Vert a_i\Vert - g \right|\right), \end{aligned}$$

where $\Xi (k) \in \{B,T,S,F\}$ describe the lower limb or exoskeleton segments, being the current Markovian state, and $\rho (k)$ is an index that describes the reliability of the accelerometer used at the Markovian state, $\Xi (k)$. The criterion to verify the reliability of the current IMU-reading is given by:

13 $$\begin{aligned} \Psi := \rho (k) \le \zeta , \end{aligned}$$

where $0< \zeta < 1$. For MJLS-based KF Algorithm refer to [6] and Additional file 1.

#### Matricial global model

In the absence of an exoskeleton, no relative sensor data can be used in the KF. In this case, for example in clinical gait analysis, cooperation between IMUs might improve the estimated absolute angles compared to local KFs. However, noise could also be introduced when the relation between the information used in the update and the segment’s orientation is less clear. It is therefore necessary to find the balance between the quantity and the quality of the performed updates. We therefore propose a matricial global KF, fusing the information of several IMUs, and evaluate it using four implementations of the threshold criterion of (15)–(18).

The threshold criterion ($\Psi$) differs among four implementations evaluated according to the amount of the reliable sensors, $\Psi _s$, $s=1,\ldots ,4$. $\Psi _s$ defined from (15) to (18). The minimum quantity of sensors that has to fulfill the threshold based criterion differs from four in (15), to one in (18). For example, if the criterion given for (16) is implemented, at least three accelerometers should be reliable to be used in the update step. In this case, one possible configuration that this criterion can assume is $\Psi = ((\rho _{B,k}< \zeta _{B}) \text {AND} (\rho _{S,k}< \zeta _{S}) \text {AND} (\rho _{F,k} < \zeta _{F}))$, with the accelerometers of the body, shank, and foot being reliable. Notice that the number of updates expected in (18) is greater than in (15). In both filters, matricial local and matricial global, these criteria are used.

14 $$\begin{aligned}\rho _{\{i,l,m\},k} & := \left(\left|\Vert a_{\{i,l,m\}}\Vert - g \right|\right) , \end{aligned}$$

15 $$\begin{aligned}\Psi _4 &:= (\rho _{B,k}< \zeta _{B}) \text {AND} (\rho _{T,k}< \zeta _{T}) \text {AND} (\rho _{S,k}< \zeta _{S}) \text {AND} (\rho _{F,k} < \zeta _{F}), \end{aligned}$$

16 $$\begin{aligned}\Psi _3 & := ((\rho _{i,k}< \zeta _{i}) \text {AND} (\rho _{l,k}< \zeta _{l}) \text {AND} (\rho _{m,k} < \zeta _{m})), \end{aligned}$$

17 $$\begin{aligned}\Psi _2 & := ((\rho _{i,k}< \zeta _{i}) \text {AND} (\rho _{l,k} < \zeta _{l})), \end{aligned}$$

18 $$\begin{aligned}\Psi _1 & := (\rho _{i,k} < \zeta _{i}), \end{aligned}$$

where $a_{\{i,l,m\}}$ are the values of each triaxial accelerometer, *g* is the earth gravity, and $\rho _{\{i,l,m\},k}$ are any reliable sensors at instant *k* being $i,l,m=\{B,T,S,F\}$ with $i \ne l \ne m$.

The matricial global model for absolute angular estimation of lower limbs segments can be described as:

19 $$\begin{aligned} \dot{x}(t)&= \bar{A} x(t) + \bar{B} w(t), \end{aligned}$$

20 $$\begin{aligned} z(t)&= \bar{C}_{c}(t) x(t) + v(t), \end{aligned}$$

where $\bar{C}_{c}(t)$ is the coupled output matrix that designs the relation between the absolute angles of the lower limb segments. The vector of output measurements is defined as:

21 $$\begin{aligned} z = \left[ \begin{array}{*{20}{c}} {\Delta \theta _{B}}&{\Delta \theta _{T}}&{\Delta \theta _{S}}&{\Delta \theta _{F}}&{\Delta \theta _{h}}&{\Delta \theta _{k}}&{\Delta \theta _{a}} \end{array}\right] ^T. \end{aligned}$$

The matrix $\bar{C}_c$ is designed in order to create relations between the four main states ($\Delta \theta _{B}$, $\Delta \theta _{T}$, $\Delta \theta _{S}$, and $\Delta \theta _{F}$), and consequently the matrix *R* must be changed. These relations are similar to joint encoders, and are presented by Eqs. (23)–(25).

22 $$\begin{aligned} \bar{C}_c \!=\! \left[ \begin{array}{*{20}{c}} 1 &{} 0 &{} 0 &{} 0 &{} 0 &{} 0 &{} 0 &{} 0\\ 0 &{} 0 &{} 1 &{} 0 &{} 0 &{} 0 &{} 0 &{} 0\\ 0 &{} 0 &{} 0 &{} 0 &{} 1 &{} 0 &{} 0 &{} 0\\ 0 &{} 0 &{} 0 &{} 0 &{} 0 &{} 0 &{} 1 &{} 0\\ 1 &{} 0 &{} { - 1} &{} 0 &{} 0 &{} 0 &{} 0 &{} 0\\ 0 &{} 0 &{} 1 &{} 0 &{} { - 1 } &{} 0 &{} 0 &{} 0\\ 0 &{} 0 &{} 0 &{} 0 &{} 1 &{} 0 &{} { - 1} &{} 0 \end{array}\right] \!\!, \;\;\; R = \left[ \begin{array}{*{20}{c}} {\sigma ^2_{\text {a}_{B}}} &{} {0} &{} {0} &{} {0} &{} {0} &{} {0} &{} {0}\\ {0} &{} {\sigma ^2_{\text {a}_{T}}} &{} {0} &{} {0} &{} {0} &{} {0} &{} {0}\\ {0} &{} {0} &{} {\sigma ^2_{\text {a}_{S}}} &{} {0} &{} {0} &{} {0} &{} {0}\\ {0} &{} {0} &{} {0} &{} {\sigma ^2_{\text {a}_{F}}} &{} {0} &{} {0} &{} {0}\\ {0} &{} {0} &{} {0} &{} {0} &{} {\sigma ^2_{\text {a}_{BT}}} &{} {0} &{} {0} \\ {0} &{} {0} &{} {0} &{} {0} &{} {0} &{} {\sigma ^2_{\text {a}_{TS}}} &{} {0} \\ {0} &{} {0} &{} {0} &{} {0} &{} {0} &{} {0} &{} {\sigma ^2_{\text {a}_{SF}}} \end{array} \right] \!\!, \end{aligned}$$

23 $$\begin{aligned} \Delta \theta _{h} = \theta _{\text {a}_{h}} - \theta _{\text {g}_{h}}, \;\;\;\; \theta _{\text {a}_{h}} = \theta _{\text {a}_{B}} - \theta _{\text {a}_{T}}, \;\;\;\; \theta _{\text {g}_{h}} = \theta _{\text {g}_{B}} - \theta _{\text {g}_{T}}, \end{aligned}$$

24 $$\begin{aligned} \Delta \theta _{k} = \theta _{\text {a}_{k}} - \theta _{\text {g}_{k}}, \;\;\;\; \theta _{\text {a}_{k}} = \theta _{\text {a}_{T}} - \theta _{\text {a}_{S}}, \;\;\;\; \theta _{\text {g}_{k}} = \theta _{\text {g}_{T}} - \theta _{\text {g}_{S}}, \end{aligned}$$

25 $$\begin{aligned} \Delta \theta _{a} = \theta _{\text {a}_{a}} - \theta _{\text {g}_{a}}, \;\;\;\; \theta _{\text {a}_{a}} = \theta _{\text {a}_{S}} - \theta _{\text {a}_{F}}, \;\;\;\; \theta _{\text {g}_{a}} = \theta _{\text {g}_{S}} - \theta _{\text {g}_{F}}. \end{aligned}$$

Additional criteria are proposed in order to verify the reliability of the cross relation between the states in matricial global approach, as can be seen:

26 $$\begin{aligned} \Upsilon _{\text {h},k} & := \left( \left( \left| \Vert a_B\Vert - g \right| \right)< \zeta _B \right) \text {AND}\left( \left( \left| \Vert a_T\Vert - g \right| \right) < \zeta _T \right) , \end{aligned}$$

27 $$\begin{aligned} \Upsilon _{\text {k},k}& := \left( \left( \left| \Vert a_T\Vert - g \right| \right)< \zeta _T \right) \text {AND}\left( \left( \left| \Vert a_S\Vert - g \right| \right) < \zeta _S \right) , \end{aligned}$$

28 $$\begin{aligned} \Upsilon _{\text {a},k} & := \left( \left( \left| \Vert a_S\Vert - g \right| \right)< \zeta _S \right) \text {AND}\left( \left( \left| \Vert a_F\Vert - g \right| \right) < \zeta _F \right) . \end{aligned}$$

$\Upsilon$ criteria are used when two segments related are found. Algorithm 1 represents a discrete KF, with matrices: $\bar{F} = I + \bar{A}T$, $\bar{G} \simeq \bar{B}T^{1/2}$, $\bar{H}_c =\bar{C}_c$.

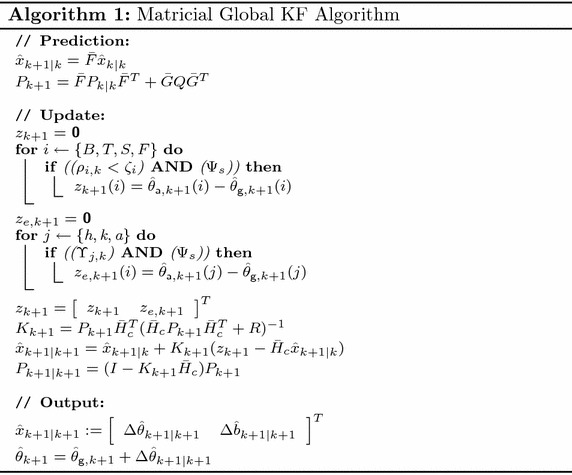



### Comparison of local and global models

Consider the matricial local model and only two segments, body (B) and thigh (T). Assume the vectors and discrete matrices in the update equations of Matricial Local KF Algorithm (see Additional file 1) to be:

$$\begin{aligned} H&= \left[ \begin{array}{llll} {1} &{} {0} &{} {0} &{} {0}\\ {0} &{} {0} &{} {1} &{} {0} \end{array} \right] , \quad \hat{x}_{k+1|k}= \left[ \begin{array}{llll} {\Delta {\hat{\theta }}_{B_{{k+1|k}}}}&{\Delta {\hat{b}}_{B_{k+1|k}}}&{\Delta {\hat{\theta }}_{T_{{k+1|k}}}}&{\Delta {\hat{b}}_{T_{k+1|k}}} \end{array} \right] ^T, \nonumber \\ z_{k+1}&= \left[ \begin{array}{l} {\Delta {\hat{\theta }}_{B_{{k+1}}}}\\ {\Delta {\hat{\theta }}_{T_{{k+1}}}} \end{array} \right] , \quad K_{k+1} = \left[ \begin{array}{llll} {k_1} &{} {k_2} &{} {k_3} &{} {k_4}\\ {k_5} &{} {k_6} &{} {k_7} &{} {k_8} \end{array} \right] ^T, \end{aligned}$$

where the weights $k_j$, for *j* = $\{1$ until $8\}$. The correction terms at any instant are:

29 $$\begin{aligned}&\hat{x}_{k+1|k+1}= \hat{x}_{k+1|k}+ K_{k+1} (z_{k+1} - H_{k+1}\hat{x}_{k+1|k}) \nonumber \\&= \left[ \begin{array}{l} {\Delta {\hat{\theta }}_{B_{{k+1|k}}}}\\ {\Delta {\hat{b}}_{B_{k+1|k}}} \\ {\Delta {\hat{\theta }}_{T_{{k+1|k}}}}\\ {\Delta {\hat{b}}_{T_{k+1|k}}} \end{array} \right] + \left[ \begin{array}{l} {k_1(\Delta {\hat{\theta }}_{B_{{k+1}}} - \Delta {\hat{\theta }}_{B_{{k+1|k}}})}\\ {k_2(\Delta {\hat{\theta }}_{B_{{k+1}}} - \Delta {\hat{\theta }}_{B_{{k+1|k}}})}\\ {k_7(\Delta {\hat{\theta }}_{T_{{k+1}}} - \Delta {\hat{\theta }}_{T_{{k+1|k}}})}\\ {k_8(\Delta {\hat{\theta }}_{T_{{k+1}}} - \Delta {\hat{\theta }}_{T_{{k+1|k}}})} \end{array} \right] + \left[ \begin{array}{l} {k_5(\Delta {\hat{\theta }}_{T_{{k+1}}} - \Delta {\hat{\theta }}_{T_{{k+1|k}}})}\\ {k_6(\Delta {\hat{\theta }}_{T_{{k+1}}} - \Delta {\hat{\theta }}_{T_{{k+1|k}}})}\\ {k_3(\Delta {\hat{\theta }}_{B_{{k+1}}} - \Delta {\hat{\theta }}_{B_{{k+1|k}}})}\\ {k_4(\Delta {\hat{\theta }}_{B_{{k+1}}} - \Delta {\hat{\theta }}_{B_{{k+1|k}}})} \end{array} \right] . \end{aligned}$$

As it can be seen in (29), the correction terms $\Delta \hat{\theta }_{B_{k+1|k+1}}$ and $\Delta \hat{\theta }_{T_{k+1|k+1}}$, for the body and thigh segments are both connected through the weights $k_j$.

However, for the matricial local model, the state-space and output equations are both diagonal or block-diagonal, the system is considered uncoupled which affects the Kalman filter gain ($K_{k+1}$) from (29). In this case $k_3$, $k_4$, $k_5$ and $k_6$ are zero. The gains $K_{k+1}$ are computed based on *P* and *H*-matrices. Since *H*, *P*, and *R* are diagonal, one can say that the $K_{k+1}$ will be mandatorily diagonal or block diagonal.

Considering the first iteration $k=0$ in the Matricial Local KF Algorithm, we have:

$$\begin{aligned} P_{1}&= \left[ \begin{array}{llll} {1} &{} {0} &{} {0} &{} {0}\\ {0} &{} {1} &{} {0} &{} {0}\\ {0} &{} {0} &{} {1} &{} {0}\\ {0} &{} {0} &{} {0} &{} {1} \end{array} \right] , \;\;\; R = \left[ \begin{array}{ll} {\sigma ^2_{\text {a}_{B}}} &{} {0} \\ {0} &{} {\sigma ^2_{\text {a}_{T}}} \\ \end{array} \right] , \\ K_{1}= & {} \left[ \begin{array}{ll} {1/\left(\sigma ^2_{\text {a}_{B}} + 1\right)} &{} {0}\\ {0} &{} {0}\\ {0} &{} {1/\left(\sigma ^2_{\text {a}_{T}} + 1\right)}\\ {0} &{} {0} \end{array} \right] , \;\;\; P_{1|1} = \left[ \begin{array}{llll} {1 - \frac{1}{\sigma ^2_{\text {a}_{B}} + 1}} &{} {0} &{} {0} &{} {0}\\ {0} &{} {1} &{} {0} &{} {0}\\ {0} &{} {0} &{} {1 - \frac{1}{\sigma ^2_{\text {a}_{T}} + 1}} &{} {0}\\ {0} &{} {0} &{} {0} &{} {1} \end{array} \right] . \end{aligned}$$

In the second iteration, $k=1$, we have:

30 $$\begin{aligned} K_{2}&= \left[ \begin{array}{ll} {-\frac{\left(\sigma ^2_{\text {a}_{B}} + 1\right)\left( \frac{1}{\sigma ^2_{\text {a}_{B}} + 1} - 1 \right) }{\sigma ^2_{\text {a}_{B}} \left(\sigma ^2_{\text {a}_{B}} + 2\right)}} &{} {0}\\ {0} &{} {0}\\ {0} &{} {-\frac{\left(\sigma ^2_{\text {a}_{T}} + 1\right)\left( \frac{1}{\sigma ^2_{\text {a}_{T}} + 1} - 1 \right) }{\sigma ^2_{\text {a}_{T}} \left(\sigma ^2_{\text {a}_{T}} + 2\right)}}\\ {0} &{} {0} \end{array} \right] . \end{aligned}$$

Again, the KF gain $K_{k+1}$ is a block diagonal matrix, and by induction it will remain block diagonal and the components $k_2$, $k_3$, $k_4$, $k_5$, $k_6$, and $k_8$ will always be zero. So, in the local matricial approach the estimated states are not connected through the components of the Kalman filter gain.

On the other hand, if there are strong relations between the states ($\hat{x}_{k+1}$) and they are not explicit in the state-space matrix *F*, the *H* matrix can be designed such that they are related. The MJLS-based and matricial global models provide this connection, and from the viewpoint of filtering the system becomes coupled and the gains of the KF will not be necessarily zero. Consider for instance the matricial global model with:

$$\begin{aligned} H = \left[ \begin{array}{llll} {1} &{} {0} &{} {0} &{} {0}\\ {0} &{} {0} &{} {1} &{} {0} \\ {1} &{} {0} &{} {-1} &{} {0} \end{array} \right] , \quad R = \left[ \begin{array}{lll} {\sigma ^2_{\text {a}_{B}}} &{} {0} &{} {0} \\ {0} &{} {\sigma ^2_{\text {a}_{T}}} &{} {0} \\ {0} &{} {0} &{} {\sigma ^2_{\text {a}_{BT}}} \\ \end{array} \right] , \end{aligned}$$

where the last line of *H* relates both segments, body and thigh. In this case, this relation can be seen as the encoder angle error. The Gaussian white noise of this relation is added to the *R* matrix being expressed by $\sigma ^2_{\text {a}_{BT}}$. Using (29), with the same $P_{k+1}$ matrix, in the first iteration $k=0$ of the Matricial Global KF Algorithm, we have

$$\begin{aligned} K_{1} = \left[ \begin{array}{lll} {1} &{} {0} &{} {1}\\ {0} &{} {0} &{} {0}\\ {0} &{} {1} &{} {-1}\\ {0} &{} {0} &{} {0} \end{array} \right] \left[ \begin{array}{lll} {\sigma ^2_{\text {a}_{B}} + 1} &{} {0} &{} {1} \\ {0} &{} {\sigma ^2_{\text {a}_{T}} + 1} &{} {-1}\\ {1} &{} {-1} &{} {\sigma ^2_{\text {a}_{BT}} + 2} \end{array} \right] ^{-1} = \left[ \begin{array}{llll} {k_1} &{} {k_2} &{} {k_3} &{} {k_4}\\ {k_5} &{} {k_6} &{} {k_7} &{} {k_8}\\ {k_9} &{} {k_{10}} &{} {k_{11}} &{} {k_{12}}\\ \end{array} \right] ^T, \end{aligned}$$

where $$k_1=\left(\sigma ^2_{\text {a}_{T}} + \sigma ^2_{\text {a}_{BT}} + \sigma ^2_{\text {a}_{T}} \sigma ^2_{\text {a}_{BT}}\right)/{D},\; k_3= \sigma ^2_{\text {a}_{T}}/{D},\; k_5={\sigma ^2_{\text {a}_{B}}}/{D}, \; k_7=\left(\sigma ^2_{\text {a}_{B}} + \sigma ^2_{\text {a}_{BT}}\left(1 + \sigma ^2_{\text {a}_{B}}\right)\right)/{D},\;k_9=\left(\sigma ^2_{\text {a}_{B}}\left(1+\sigma ^2_{\text {a}_{T}}\right) - 1\right)/D, \; k_{11}=-\left(\sigma ^2_{\text {a}_{T}}\left(1+\sigma ^2_{\text {a}_{B}}\right) - 1\right)/D, \; D=\sigma ^2_{\text {a}_{B}} \left(1 + 2 \sigma ^2_{\text {a}_{T}} + \sigma ^2_{\text {a}_{BT}} + \sigma ^2_{\text {a}_{T}} \sigma ^2_{\text {a}_{BT}}\right) + \sigma ^2_{\text {a}_{T}} + \sigma ^2_{\text {a}_{BT}} \left(1 + \sigma ^2_{\text {a}_{T}}\right).$$

The remaining gains are all zero ($k_2 = k_4 = k_6 = k_8 = k_{10} = k_{12} = 0$). So, already in the first iteration, the gains $k_3$ and $k_5$ directly relate the states ($\Delta \theta _{B}$ and $\Delta \theta _{T}$), and the gains $k_9$ and $k_{11}$ represent the cross relation between them. Moving to the second iteration $k = 1$, and considering that *P* was updated, we will have again the same gains $K_{k+1}$ different than zero. In other words, the states will be corrected by a weighting of the correction terms of both segments in the KF.

### Experimental setup

Data from a single trial of a healthy male subject (age: 33; height: 1m75; weight: 82 kg) experienced in walking with an exoskeleton are presented. The trial consisted of walking on a treadmill at 1.5 km/h for one minute. Kinematic data were obtained from three independent systems, synchronized using an electronic trigger, see Fig. 1.

An optoelectronic system (BTS Bioengineering) was used to produce the reference measurement of absolute segment orientation. Clusters of markers were attached to the segments and sampled at 100 Hz. For each segment one cluster of three markers was used to compute the orientation of that segment.

Four IMUs were attached to the lateral side of the right shank, thigh and the body, and on top of the right foot aligned with the sagittal plane and the longitudinal axis of the foot, see Fig. 1. The inertial sensors used are the TechMCS IMUs (Technaid SL, Spain). IMU data were acquired in digital format at 50 Hz, and transformed offline to physical format. Both the markers and the inertial sensors are attached to the same rigid plate, and aligned to both the exoskeleton and the subject in the sagittal plane.

The exoskeleton used is the H2 [21], a bilateral exoskeleton with six actuated degrees of freedom at the hip, knee, and ankle sagittal plane joints of both legs. Relative joint angles are measured by the H2 using precision industrial potentiometers with a linearity of 0.25% [21]. Potentiometer data were acquired at 1 kHz.

**Fig. 2:**
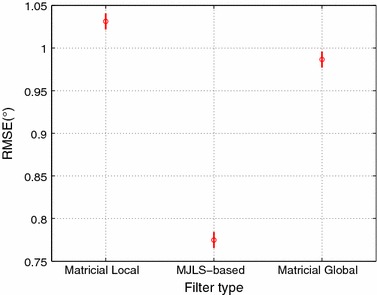
Pos-hoc LSD test, comparing the main effect of filters proposed

All data were resampled at 50 Hz. The first and last 10 s of the trial were discarded. The remaining 40 seconds were divided into two blocks. The first 20 s were used for optimization of the filter parameters. The remaining 20 s were used for validation of the estimated absolute angles. As the KFs convergence has been demonstrated to be sensitive to the starting point, fifty different starting points were used both in the optimization and validation stage, [22]. The first and last intervals of the validation stage are indicated by horizontal lines in Fig. 2. Filter parameters were optimized using a genetic algorithm [18].

### Data analysis

To assess the effects of the filter used on the estimates of the absolute angle of each segment, a four-way analysis of variance (ANOVA) with significance level at 5% was performed with the root mean square error (RMSE) as dependent variable,

31 $$\begin{aligned} RMSE_i = \sqrt{\frac{1}{N} \sum _{k=1}^N (\hat{\theta }_i - \theta _{i_{ref}})^2}. \end{aligned}$$

The four factors were: filter, segment, start point, and samples. A post-hoc Tukey’s Least Significant Difference (LSD) testing was performed when needed. All the data processing and statistical analysis were done using custom scripts in Matlab (The Mathworks, USA).

## Results

**Table 1:** Performance index (RMSE) for the filters

Filters type			
Segm.	Matricial local	MJLS-based	Matricial global
Body	1.923	0.775	1.964
Thigh	0.772	0.779	0.664
Shank	0.660	0.768	0.568
Foot	1.453	1.447	1.470
Average	1.202	0.942	1.167
Acc.Rel.	36.1%	21.7%	22.2%

**Table 2:** Performance index for the matricial global ($\gamma$C) and local ($\gamma$UC) filters, with $\gamma$ varying between 1 and 4 reliable accelerometers, in which, each of the criteria [$\Psi$ in (15)–(18), and $\Upsilon$ in (26)–(28)] are applied in the matricial filters algorithms

Segments	Filters criteria							
	4C	4UC	3C	3UC	2C	2UC	1C	**1UC**
Body	1.649	1.684	1.980	2.063	1.964	1.923	2.191	2.335
Thigh	0.926	0.937	0.652	0.702	0.664	0.772	0.815	0.810
Shank	0.783	0.818	0.858	0.956	0.568	0.660	0.606	1.656
Foot	1.456	1.439	1.618	1.586	1.470	1.453	1.359	1.316
Average	1.204	1.220	1.277	1.327	1.167	1.202	1.243	1.529
AR (%)	2.4	2.4	11.3	14.3	22.2	36.1	29.2	64
AGR (%)	2.4	–	9.4	–	15	–	15	–

The accelerometer reliability (AR) and accelerometer global reliability (AGR) are also shown

Table 1 summarizes the RMSE between the absolute angles obtained from the optic system and each filter, as well as the amount of time (expressed in percentage of total trial duration) the accelerometer data are used to update the filter. Since all but the least stringent matricial (local/global) KFs performed similarly, see Table 2, we only use the matricial KFs based on the criterion (17), in which two or more IMUs have to be reliable in order to be used in the update. As can be seen from Table 1, the global KF solutions achieved a higher accuracy than the local KF, with an average accuracy of 1.202° for the local KF, 0.942 for the MJLS-based KF, and 1.167 for the matricial global KF. The matricial global performs best for the shank and thigh segments, but offers little to no improvement on the body when compared to the local solution. The MJLS-based KF performs similarly to the matricial global on all segments but the body, where vastly outperforms the other solutions on the body, resulting in an overall best performance for the MJLS-based.

**Fig. 3:**
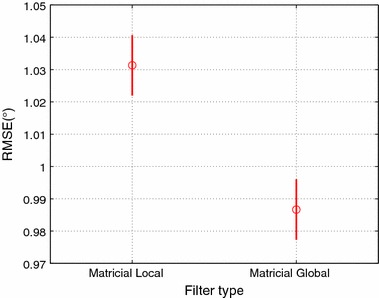
Pos-hoc LSD test, comparing the main effect of Matricial Local and Matricial Global filters

**Fig. 4:**
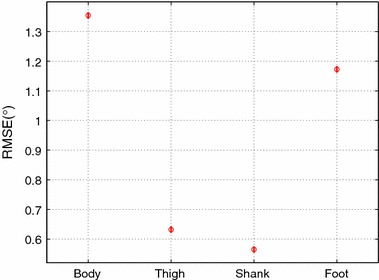
Pos-hoc LSD test, comparing the main effect of the filters through the limb segments dimension

**Fig. 5:**
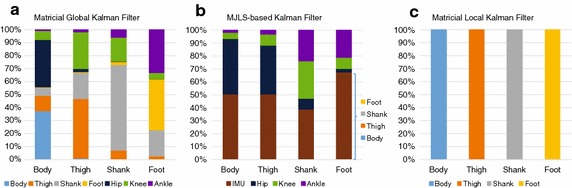
Graphical representation of the cross-collaboration by each term of the KFs gain  $K_{k+1}$. Used in KF Algorithms (see Additional file 1 and Algorithm 1), with: **a** matricial global, **b** Markovian, **c** matricial local Kalman filters

The statistical analysis shows that there are significant differences in the filters ($F=404.78$ and $p=0.0000$) and segments ($F=2480.84$ and $p=0.0000$) dimensions. The Post-hoc Tukey’s LSD demonstrated that, in the filters dimension, the MJLS-based performed better than the other filters, see Fig. 3 and Table 1. Repeating the statistical analysis for the matricial global/local approaches, it shows significant differences between these filters ($F=21.89$ and $p=0.0000$). The LSD demonstrated that the matricial global performed better than matricial local, see Fig. 4. Furthermore, in segments dimension, the LSD has shown that the body and foot have the worst performance, and no big difference was observed between the thigh and shank, see Fig. 5.

**Fig. 6:**
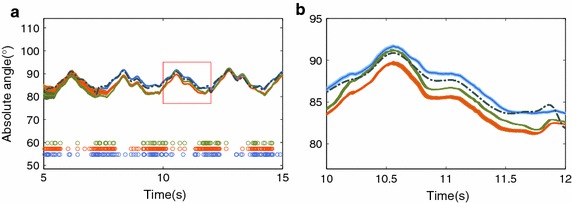
Absolute body angle with all filters. Where the colors *olive*, *orange* and *blue* represent respectively the approaches: matrix global, matrix local both with $\gamma = 2$ and MJLS-based KF. The *circles* are the instants in which the criteria $\Psi$ were satisfied

Figure 6a breaks down how the information is used by each segment in the matricial global filter. For example, in the first bar, representing the body, the information obtained directly from the IMU attached to the body makes up 38% (light blue section), the information obtained directly from the IMUs attached to the other segments represents around 20% (orange, grey and yellow sections combined), the remaining 42% is obtained from the relation between the segments. This is not measured directly but represented mathematically through the weights given to the residual error of the relative angle between segments. In the example of the body (first bar of Fig. 6a) the relative angle that contributes most is the hip (dark blue, around 37%). In Fig. 6b, the body segment is estimated by the MJLS-based KF based on information from the IMU (brown section) and the relation between the segments, provided by the encoder data. In the MJLS-based KF this relation is represented by the mechanical structure of the exoskeleton and captured directly by the encoders. In the matricial global this relation is mathematical and derived indirectly by computing the angle between segments. The stronger relation in the MJLS-based KF appears to be particularly helpful for the estimation of the the hip joint movement. In Fig. 6c the local case is shown where no information is shared between the segments.

In the MJLS-based model, the Markovian jumps are used to decide which IMU is used to complement the encoder information. Despite all IMUs being eligible for use in a filter update, the IMU attached to the body is never used by the MJLS-based KF.

**Fig. 7:**
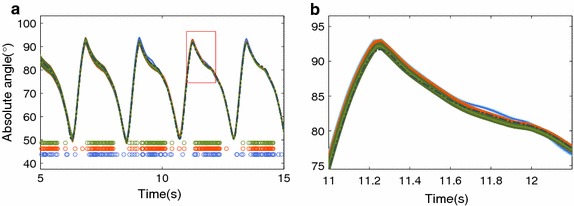
Absolute shank angle with all filters. Where the colors *olive*, *orange* and *blue* represent respectively the approaches: matrix global, matrix local both with $\gamma = 2$ and MJLS-based KF. The *circles* are instants fulfilling the criteria $\Psi$

**Fig. 8:**
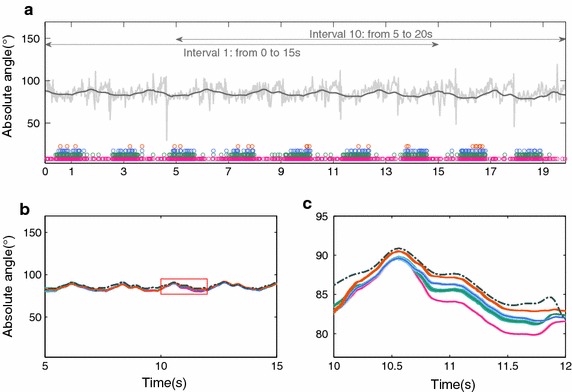
Absolute body angle of the matricial local model. Where in (**a**) the *gray* and *dark gray* signals are respectively the accelerometer and gyroscope data. The *circles* are instants fulfilling the criterion $\Psi$ with matrix approach, where the colors *orange*, *blue*, *green* and *pink* represent $\Psi$ being defined respectively by Eqs. (15) to (18). **b** and **c** are the absolute body angles estimation

In contrast to the matricial local KF, both global KFs show a better spread of accelerometer based updates, see Figs. 7 and 8.The local KF does update more often (higher total number of updates) than any of the global solutions presented.

**Fig. 9:**
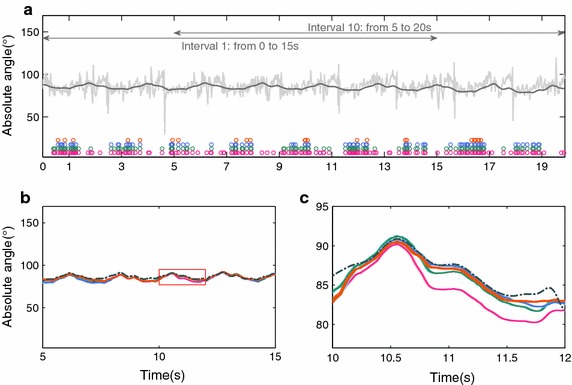
Absolute body angle of the matricial global. Where in **a** the *gray* and *dark gray* signals are respectively the accelerometer and gyroscope data. The *circles* are instants fulfilling the criterion $\Psi$ with matrix approach, where the colors *orange*, *blue*, *green* and *pink* represent $\Psi$ being defined respectively by Eqs. (15) to (18). The sub-figures (**b**) and (**c**) are the absolute body angles estimation

Table 2 summarizes the RMSE for the matricial filters (global and local). The accelerometer reliability indicates the percentage of the trial duration when the accelerometers are used to update the estimated absolute angles. A more rigorous criterion resulted in fewer updates and lower accelerometer reliability, see Figs. 2 and 9. Interestingly, with this implementation the updates are fewer and only occur around the stance phase. This criterion results in the use of accelerometers data for only 2.4% of the trial duration, for both matricial filters. On the other hand, the most lenient criterion, (18), updates the estimate in 29.7 and 65.3% for global and local matricial filters respectively. Accelerometer coupled reliabilities, $\Upsilon$ in (26)–(28), indicate the percentage of the trial duration with coupled accelerometers used together to update the estimated absolute angles, Table 2.

Despite the global filter updating on fewer moments than the local filter, it produces better results in most cases. For example, with the criterion (18), in Table 2 one can see that the matricial global filter shows an error of $1.243^{\circ }$, using accelerometer data for 29.2% of the trial, and the accelerometer coupled reliability being 15% of the trial. We can also appreciate that matricial local filter shows an error of $1.529^{\circ }$, using accelerometer data during 64% of the trial. This suggests the ability of the global filters to take advantage of the relationships between the states expressed in the global model.

**Fig. 10:**
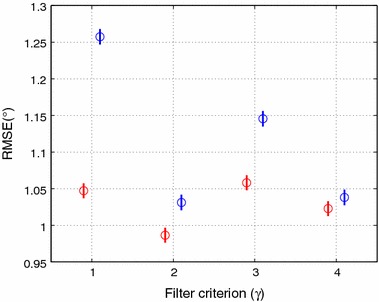
Pos-hoc LSD test, comparing matricial global with matricial local filters. Where *red color* represent the matricial global and the *blue color* the matricial local. The filter criterion $\gamma$ assuming 1–4 are respectively the Eqs. (18) to (15)

The RMSE values in Table 2 shows that all but the most lenient implementation result in a similar performance. Statistical analysis revealed that, for the local filter, the usage of the criteria (16) and (18), differed significantly from the other two matricial KF implementations ($F=194.15$ and $p=0.0000$). No significant differences were found among the other implementations, Fig. 10 enhances these, where the confidence intervals for each implementation is shown in blue. The same analysis, for the global filter, revealed that the criterion (17) differed significantly from the other three matricial global KF implementations ($F=18.58$ and $p=0.0000$), despite the matricial global KFs performance appear to be less sensitive to the criterion used. No significant differences were found among the other implementations criteria, see Fig. 10.

Figures 2 and 9 shows the corresponding absolute angle of the body, for both local and global matricial filters. For the most stringent criterion the estimate is in better agreement with the reference. These results suggest that the quality of the update is more important than the quantity. This is also shown in Figs. 2c and 9c where, despite the frequent updates, the least stringent implementation is unable to converge to the reference at a faster rate than the other implementations.

The results for the thigh and shank are better than those for the body and foot, with RMSE values most of time smaller than 1 for all but the least strict implementation. Nonetheless, a trend towards better performance can also be observed as the criterion becomes more stringent. Fig. 10 reports the results of the difference matricial KF implementations for each segment are included in the online appendix (see Additional file 2). The statistical analysis shows that the performance of the matricial filters, local and global, are strongly dependent on the segment ($F=3726.16$ and $p=0.0000$) and ($F=4317.77$ and $p=0.0000$).

## Discussion

The body is the only segment that was not used in the MJLS-based KF updates, since it never achieves the criteria performance Eqs. (12) and (13). This means that in MJLS-based KF the residual error used to estimate the absolute angles for each segment does not include the residual angle error of the body segment. However, the segment with the best improvement by the MJLS-based KF was the body segment, which demonstrate the strong influence of the this global approach. The MJLS-based appears to achieve this better performance through a high number of updates with only the best inertial sensor signal at each instant.

The matricial global KF proposed and validated in this paper, as well as the MJLS-based KF proposed in [6], outperformed the local KF (Table 1). Mean RMSE dropped from 1.202 for the local KF to 1.167 for the matricial global KF, and 0.942 for the MJLS-based KF. It is thus beneficial to take advantage of the information present in other segments and the relation between segments. Fig. 6 clarifies just how big the contribution of this indirect information is. This is also the only difference between the matricial global and the matricial local filters.

In the MJLS-based KF this relation is more explicit and measured directly by the potentiometers embedded in the exoskeleton. Surprisingly, no real differences in terms of the RMSE at the level of the thigh and shank were found between the MJLS-based and the matricial global KFs. The MJLS-based KF did vastly outperform the matricial global KF for the absolute orientation of the body.

The relative joint angle is a main contributor to determine body orientation (Fig. 6a, b. Likely the potentiometer data is of higher quality, partly explaining why the MJLS-based KF performed much better at the body level.

The best combination of sensors based on the criteria $\Psi$ and $\Upsilon$ used in the KF updates likely depends on the task. In this paper, data from a gait trial were analyzed. Results in terms of RMSE and the statistical analysis were similar across the diverse implementations of the matricial global KFs tested in this work. This might be due to the rather slow walking speed, 1.5 m/s, and the cyclic nature of gait. For other tasks a clearer difference might exist. The matricial global and MJLS-based KFs differ in the information that is fused in their models. The matricial global KF only relies on IMU data, and can thus also be applied to clinical gait analysis or gait monitoring. The MJLS-based relies on the information from the potentiometers embedded in the exoskeleton, and is therefore suited to be embedded in the control algorithm.

The information from the potentiometer data, the joint angles, thus proved to be more useful than the additional information from the remaining IMUs. In the matricial global KF, the relation between the IMUs is incorporated in the model through the output matrices. The better performance of the MJLS-based compared to the matricial global KF might be due to the more reliable relation between segments when using the relative angles from the potentiometers, compared to the relative angles obtained through IMUs. Additionally, the fact that in the MJLS-based only the most reliable accelerometer is used to estimate the absolute angles may have contributed to the observed improvement.

In the Fig. 6 was shown the strong influence of the KF gain in global models, where in most cases of Fig. 6a more than 50% of the information originates from the relation between segments. In Fig. 6b with exception from the foot, the encoder data is the dominant information to determine segment orientation.

In the MJLS-based the foot segment was the only segment with RMSE greater than 1°, Table 1. This might have been due to a less optimal fit between the subject’s foot and the exoskeleton, as well as the fitting of the sagittal plane exoskeleton to the ankle. Unlike the knee, the ankle has substantial movement and range of movement outside of the sagittal plane.

**Fig. 11:**
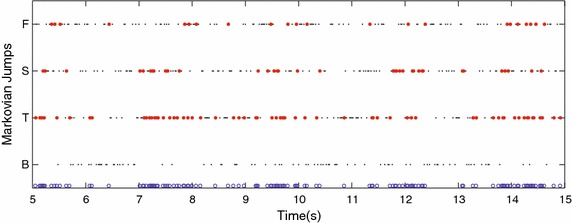
Markov chain jumps applied by the MJLS-based KF. This figure shows which IMU was used, how often and when. The *black dots* are the Markovian states, *red dots* represent Markovian states fulfilling the criterion $\Psi$, the *blue circles* are the summary of all Markovian states with reliable data

The hinge joint of the exoskeleton used and most widely available exoskeleton restricting motion with a functional advantage in many cases, but the method is still valid. The body or pelvis is currently the most common location to place an inertial sensor to obtain absolute orientation of an exoskeleton. Our results indicate that the best options would be the thigh or shank segments, since they have more instants in which the accelerometers are reliable (Fig. 11), and the accuracy achieved in matricial local KF is greater for these segments (Table 2). The effect of this is also present in the matricial global KF. These results thus indicate that if the only goal of the sensor is to obtain absolute orientation of the exoskeleton, that it is best to not place it at the body.

Future work should consider exoskeletons with a more physiological ankle joint for the MJLS-based. The performance of the presented filters was segment dependent, a hybrid approach should therefore be considered with a different solution for the end-segments of the exoskeleton’s chain (body and foot). Furthermore, additional trials should be done on pathological gait and over a more extensive dataset.

## Conclusion

We presented and validated a novel global KF, and validated the MJLS-based KF [6] previously presented by the authors. Both global filters outperformed the commonly used local KF. Our results thus suggest that global KF solutions are superior to local KF solutions for gait analysis. The MJLS-based KF performed best, in particular due to a better performance at the body level. In the MJLS-based KF a direct measurement of relative angles between segments is available and exploited. In the presented matricial global KF only an indirect measurement, based on the absolute angle of each segment can be used. This paper has shown that even when only this indirect measurement is available, significant improvements in accuracy are obtained by filtering globally compared to using local filter models. We verified the influence of the criterion used to determine the accelerometer updates and found that the presented global filter performed similarly, although slightly better with more stringent criteria applied. The local KF performance varied greatly depending on the criteria used,but overall improved when the criteria became more stringent. In all cases the global solution performed better, further underlining the importance of the information present in the other segments and in the relation between those segments. We therefore recommend the use of global KFs for the analysis of lower limb motion, in particular for gait analysis and control.

## Additional files

**Additional file 1.** Mathematical models.

**Additional file 2.** Results report.

## Abbreviations

KF: Kalman filter
MJLS: Markov jump linear systems
RMSE: Root mean square error
IMU: Inertial measurement unit
MIMU: Magnetic and inertial measurement unit
ANOVA: Analysis of variance
LSD: Least significant difference
