# Supplementary material for: Global Kalman filter approaches to estimate absolute angles of lower limb segments
Source: Biomed Eng Online. 2017 May 16;16:58. doi: 10.1186/s12938-017-0346-7 (PMC5434567; doi:10.1186/s12938-017-0346-7)
Supplement: Supplementary file 1 — Additional file 1. Mathematical models. [file 12938_2017_346_MOESM1_ESM.pdf]

Mathematical Models

1 General description

In this supplementary material are shown the Matricial local and Markovian model in more details.

1.1 Matricial local model

The local models equations are formulated in matrix arrangement, where the state-space and output equations can be summarized as:

$$\dot{x}(t) = \bar{A}x(t) + \bar{B}w(t), \quad (1)$$

$$z(t) = \bar{C}(t)x(t) + v(t), \quad (2)$$

the state vector is defined as $x = \left[\begin{array}{cccc} x_B & x_T & x_S & x_F \end{array}\right]^T$, with $x_i = \left[\begin{array}{cc} \Delta\theta_i & \Delta b_i \end{array}\right]$; $\Delta\theta_i = \theta_i - \hat{\theta}_{i_g}$, are the errors between the absolute angles (θ_i) and the angle estimates calculated by the gyroscopes ($\hat{\theta}_{i_g}$); and Δb_i, are the errors of the bias generated by the gyroscopes for each segment, for $i = \{B, T, S, F\}$, where B, T, S, and F stand for body, thigh, shank, and foot segments, respectively. The vectors $w(t)$ and $v(t)$ contain the white noise Gaussian of the gyroscopes, gyroscope bias, and accelerometer respectively.

Considering the gyroscope and gyroscope bias models, the matrices $\bar{A}$

and $\bar{C}$ are defined as:

$$\bar{A} = \begin{bmatrix} 0 & 1 & 0 & 0 & 0 & 0 & 0 & 0 \\ 0 & -1/\tau_{g_B} & 0 & 0 & 0 & 0 & 0 & 0 \\ 0 & 0 & 0 & 1 & 0 & 0 & 0 & 0 \\ 0 & 0 & 0 & -1/\tau_{g_T} & 0 & 0 & 0 & 0 \\ 0 & 0 & 0 & 0 & 0 & 1 & 0 & 0 \\ 0 & 0 & 0 & 0 & 0 & -1/\tau_{g_S} & 0 & 0 \\ 0 & 0 & 0 & 0 & 0 & 0 & 0 & 1 \\ 0 & 0 & 0 & 0 & 0 & 0 & 0 & -1/\tau_{g_F} \end{bmatrix}, \quad \bar{C} = \begin{bmatrix} 1\,0\,0\,0\,0\,0\,0\,0 \\ 0\,0\,1\,0\,0\,0\,0\,0 \\ 0\,0\,0\,0\,1\,0\,0\,0 \\ 0\,0\,0\,0\,0\,0\,1\,0 \end{bmatrix} \quad (3)$$

which produce the output vector $z = \begin{bmatrix} \Delta\theta_B & \Delta\theta_T & \Delta\theta_S & \Delta\theta_F \end{bmatrix}$. $\bar{B}$ is an identity matrix.

The weighting matrices Q and R are time-invariant and given by:

$$Q = \begin{bmatrix} \sigma^2_{g_B} & 0 & 0 & 0 & 0 & 0 & 0 & 0 \\ 0 & \sigma^2_{b_{g_B}} & 0 & 0 & 0 & 0 & 0 & 0 \\ 0 & 0 & \sigma^2_{g_T} & 0 & 0 & 0 & 0 & 0 \\ 0 & 0 & 0 & \sigma^2_{b_{g_T}} & 0 & 0 & 0 & 0 \\ 0 & 0 & 0 & 0 & \sigma^2_{g_S} & 0 & 0 & 0 \\ 0 & 0 & 0 & 0 & 0 & \sigma^2_{b_{g_S}} & 0 & 0 \\ 0 & 0 & 0 & 0 & 0 & 0 & \sigma^2_{g_F} & 0 \\ 0 & 0 & 0 & 0 & 0 & 0 & 0 & \sigma^2_{b_{g_F}} \end{bmatrix}, \quad R = \begin{bmatrix} \sigma^2_{a_B} & 0 & 0 & 0 \\ 0 & \sigma^2_{a_T} & 0 & 0 \\ 0 & 0 & \sigma^2_{a_S} & 0 \\ 0 & 0 & 0 & \sigma^2_{a_F} \end{bmatrix} \quad (4)$$

where $\sigma^2_{g_i}$ and $\sigma^2_{b_{g_i}}$ are the variances of the gyroscope and gyroscope bias white Gaussian noise, respectively.

The criterion Ψ for the *matricial uncoupled* based differs among four implementations evaluated according to the amount of the reliable sensors, Ψ_s, $s = 1, , 4$. Ψ_s defined from (6) to (9). The minimum quantity of sensors that has to fulfill the threshold based criterion differs from four in (6), to one in (9). For example, if the criterion given for (7) is implemented, at least three accelerometers should be reliable to be used in the update step. In this case, one possible configuration that this criterion can assume is $\Psi = ((\rho_{B,k} < \zeta_B)\text{AND}(\rho_{S,k} < \zeta_S)\text{AND}(\rho_{F,k} < \zeta_F))$, with the accelerometers of the body, shank, and foot being reliable. Notice that the number of

updates expected in (6) is greater than (9).

$$\rho_{\{i,l,m\},k} := \left(\left|\|a_{\{i,l,m\}}\| - g\right|\right), \tag{5}$$

$$\Psi_4 := (\rho_{B,k} < \zeta_B)\text{AND}(\rho_{T,k} < \zeta_T)\text{AND}(\rho_{S,k} < \zeta_S)\text{AND}(\rho_{F,k} < \zeta_F), \tag{6}$$

$$\Psi_3 := ((\rho_{i,k} < \zeta_i)\text{AND}(\rho_{l,k} < \zeta_l)\text{AND}(\rho_{m,k} < \zeta_m)), \tag{7}$$

$$\Psi_2 := ((\rho_{i,k} < \zeta_i)\text{AND}(\rho_{l,k} < \zeta_l)), \tag{8}$$

$$\Psi_1 := (\rho_{i,k} < \zeta_i), \tag{9}$$

where $a_{\{i,l,m\}}$ are the values of each triaxial accelerometer, g is the earth gravity, and $\rho_{\{i,l,m\},k}$ are any reliable sensors at instant k being $i, l, m = \{B, T, S, F\}$ with $i \neq l \neq m$.

Therefore, the recursive *prediction* and *update* equations for the matricial local KF are presented here in Algorithm 1, with matrices: $\bar{F} = I + \bar{A}T$, $\bar{G} \simeq \bar{B}T^{1/2}$, $\bar{H} = \bar{C}_c$.

Algorithm 1: Matricial Local KF Algorithm

// **Prediction:**

$\hat{x}_{k+1|k} = \bar{F}\hat{x}_{k|k}$

$P_{k+1} = \bar{F}P_{k|k}\bar{F}^T + \bar{G}Q\bar{G}^T$

// **Update:**

$z_{k+1} = \boldsymbol{0}$

for $i \leftarrow \{B, T, S, F\}$ **do**

  if *(($\rho_{i,k} < \zeta_i$)* **AND** *(Ψ_s))* **then**

    $z_{i,k+1} = \hat{\theta}_{\text{a},k+1}(i) - \hat{\theta}_{\text{g},k+1}(i)$

$K_{k+1} = P_{k+1}\bar{H}^T(\bar{H}P_{k+1}\bar{H}^T + R)^{-1}$

$\hat{x}_{k+1|k+1} = \hat{x}_{k+1|k} + K_{k+1}(z_{k+1} - \bar{H}\hat{x}_{k+1|k})$

$P_{k+1|k+1} = (I - K_{k+1}\bar{H})P_{k+1}$

// **Output:**

$\hat{x}_{k+1|k+1} := \left[\begin{array}{cc} \Delta\hat{\theta}_{k+1|k+1} & \Delta\hat{b}_{k+1|k+1} \end{array}\right]^T$

$\hat{\theta}_{k+1} = \hat{\theta}_{\text{g},k+1} + \Delta\hat{\theta}_{k+1|k+1}$

1.2 MJLS-based model

The Markovian Jump Linear System-based model presented in [1] fuses inertial sensors with relative joint sensors embedded in the H2 exoskeleton [2]. The correction terms ($\Delta\hat{\theta}_{i_{k+1|k+1}}$) contain a weighting of the most reliable IMU and the additional terms are based on the potentiometers, see (12) and (17). Only the IMU that best meets the threshold criterion is used. We provide a brief overview of the algorithm below, but refer to [1] and the online supplementary material [see Addition file 2, Section 1.2] for a more detailed description.

The MJLS-model model for absolute angular estimation of lower limb exoskeletons can be described by the following state-space equations:

$$\dot{x}(t) = \bar{A}x(t) + \bar{B}w(t), \tag{10}$$

$$z(t) = \bar{C}_{\Xi}(t)x(t) + v(t), \tag{11}$$

where $\Xi(t) \in \{B, T, S, F\}$ defines the possible Markovian jumps. The vector of output measurements is defined as:

$$z = \left[\ \Delta_{IMU}\ \ \Delta\theta_h\ \ \Delta\theta_k\ \ \Delta\theta_a\ \right]^T, \tag{12}$$

where $\Delta_{IMU} = \left[\ \hat{\theta}_{\mathrm{a}_{IMU}} - \hat{\theta}_{\mathrm{g}_{IMU}}\ \right]$ are the errors between the estimates of the absolute angles calculated for each segment by the accelerometers ($\hat{\theta}_{\mathrm{a}_{IMU}}$) and the estimates calculated by the gyroscopes ($\hat{\theta}_{\mathrm{g}_{IMU}}$); and $\Delta\theta_j = \left[\ \Delta\theta_i - \Delta\theta_{i+1}\ \right]$, for $j = \{h, k, a\}$, are the errors of the relative angles of the corresponding joints, j, where h, k and a stand for hip, knee, and ankle.

The gyroscope, accelerometer and gyroscope bias, are modeled by matrices $\bar{A}$ and $\bar{B}$ which are defined as in Algorithm Local KF, using a matrix arrangement of the local models equations for each segment. Matrix $\bar{C}$ is defined as:

$$\bar{C}_{\Xi}(t) = \begin{bmatrix} M_B(t) & 0 & M_T(t) & 0 & M_S(t) & 0 & M_F(t) & 0 \\ 1 & 0 & -1 & 0 & 0 & 0 & 0 & 0 \\ 0 & 0 & 1 & 0 & -1 & 0 & 0 & 0 \\ 0 & 0 & 0 & 0 & 1 & 0 & -1 & 0 \end{bmatrix} \tag{13}$$

where M_i, for $i = \{B, T, S, F\}$, assumes values of zero or one, according to the angle associated with the value of Δ_{IMU}. The time-varying matrix $R_{\Xi(t)}$

is defined as:

$$R_{\Xi(t)} = \begin{bmatrix} \sigma^2_{\mathrm{a}_{\Xi(t)}} & 0 & 0 & 0 \\ 0 & \sigma^2_{\mathrm{e}_h} & 0 & 0 \\ 0 & 0 & \sigma^2_{\mathrm{e}_k} & 0 \\ 0 & 0 & 0 & \sigma^2_{\mathrm{e}_a} \end{bmatrix}, \tag{14}$$

where $\sigma^2_{\mathrm{a}_{\Xi(t)}}$ is the variance of the most reliable accelerometer at instant t; $\sigma^2_{\mathrm{e}_h}$, $\sigma^2_{\mathrm{e}_k}$, and $\sigma^2_{\mathrm{e}_a}$ are the variances of relative joint sensors fixed in hip, knee, and ankle joints, respectively. The criteria for reliable accelerometer measurements and the Markovian state in discrete time are defined as:

$$\rho(k) := \min_i \left(\left| \|a_i\| - g \right| \right), \tag{15}$$

$$\Xi(k) := \arg\min_i \left(\left| \|a_i\| - g \right| \right), \tag{16}$$

where $\Xi(k) \in \{B, T, S, F\}$ describe the lower limb or exoskeleton segments, being the current Markovian state, and $\rho(k)$ is an index that describes the reliability of the accelerometer used at the Markovian state, $\Xi(k)$. The criterion to verify the reliability of the current IMU-reading is given by:

$$\Psi := \rho(k) \leq \zeta, \tag{17}$$

where $0 < \zeta < 1$. The MJLS-based KF is by Algorithm 2, with matrices: $\bar{F} = I + \bar{A}T$, $\bar{G} \simeq \bar{B}T^{1/2}$, $\bar{H}_{\Xi(k)} = \bar{C}_{\Xi(k)}$.

Algorithm 2: MJLS-based KF Algorithm

// **Prediction:**

$\hat{x}_{k+1|k} = \bar{F}\hat{x}_{k|k}$

$P_{k+1} = \bar{F}P_{k|k}\bar{F}^T + \bar{G}Q\bar{G}^T$

// **Update:**

$z_{\Xi(k),k+1} = \boldsymbol{0}$

if (Ψ) **then**

  $z_{\Xi(k),k+1} = \hat{\theta}_{\mathrm{a},k+1} - \hat{\theta}_{\mathrm{g},k+1}$

$z_{e,k+1} = \theta_{\mathrm{e},k+1} - \hat{\theta}_{\mathrm{e},k+1}$

$z_{k+1} = \begin{bmatrix} z_{\Xi(k),k+1} & z_{e,k+1} \end{bmatrix}^T$

$K_{k+1} = P_{k+1}\bar{H}^T_{\Xi(k)}(H_{\Xi(k)}P_{k+1}\bar{H}^T_{\Xi(k)} + R_{\Xi(k)})^{-1}$

$\hat{x}_{k+1|k+1} = \hat{x}_{k+1|k} + K_{k+1}(z_{k+1} - \bar{H}_{\Xi(k)}\hat{x}_{k+1|k})$

$P_{k+1|k+1} = (I - K_{k+1}\bar{H}_{\Xi(k)})P_{k+1}$

// **Output**

$\hat{x}_{k+1|k+1} := \begin{bmatrix} \Delta\hat{\theta}_{k+1|k+1} & \Delta\hat{b}_{k+1|k+1} \end{bmatrix}^T$

$\hat{\theta}_{k+1} = \hat{\theta}_{\mathrm{g},k+1} + \Delta\hat{\theta}_{k+1|k+1}$

References

[1] Nogueira, S.L., Siqueira, A.A.G., Inoue, R.S., Terra, M.H.: Markov jump linear systems-based position estimation for lower limb exoskeletons. Sensors **14**(1), 1835–1849 (2014). doi:10.3390/s140101835

[2] Bortole, M., del Ama, A., Rocon, E., Moreno, J.C., Brunetti, F., Pons, J.L.: A robotic exoskeleton for overground gait rehabilitation. In: Robotics and Automation (ICRA), 2013 IEEE International Conference On, pp. 3356–3361 (2013). doi:10.1109/ICRA.2013.6631045
