# Supplementary material for: Global Kalman filter approaches to estimate absolute angles of lower limb segments
Source: Biomed Eng Online. 2017 May 16;16:58. doi: 10.1186/s12938-017-0346-7 (PMC5434567; doi:10.1186/s12938-017-0346-7)
Supplement: Supplementary file 2 — Additional file 2. Results report. [file 12938_2017_346_MOESM2_ESM.pdf]

Results report

Local and Global KF results

In this additional material are shown the results for all segments using local and global Kalman filter based algorithms. In Sections 1 and 2 will be present results for the local and global matricial filters for the different type of the criterion Ψ. Already in Section 3 will be present results comparing the local and global filters.

1 Matricial Local KF results

In this section are shown the results for the matricial local KF.

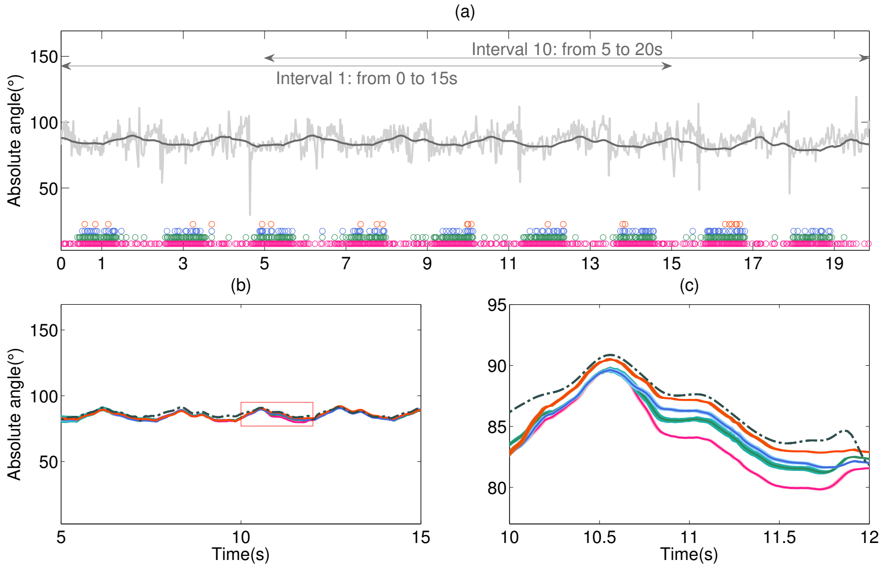

Figure 1: **Absolute trunk angle of the Matricial Local Model**. Where in (a) the gray and dark gray signals are respectively the accelerometer and gyroscope data. The circles are instants fulfilling the criterion Ψ with matrix approach, where the colors orange, blue, green and pink represent Ψ being defined respectively by equations (15) to (18) from the paper. The sub-figures (b) and (c) are the absolute trunk angles estimation.

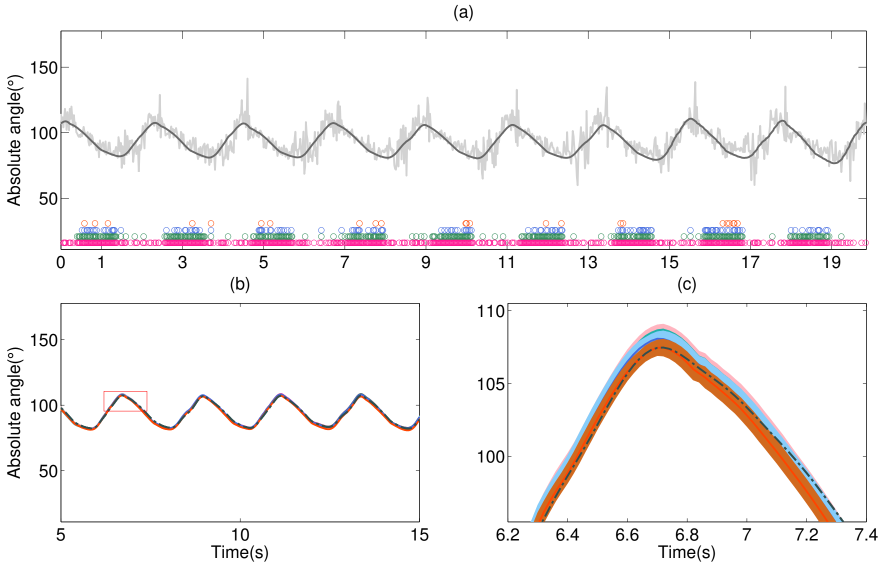

Figure 2: **Absolute thigh angle of the Matricial Local Model**

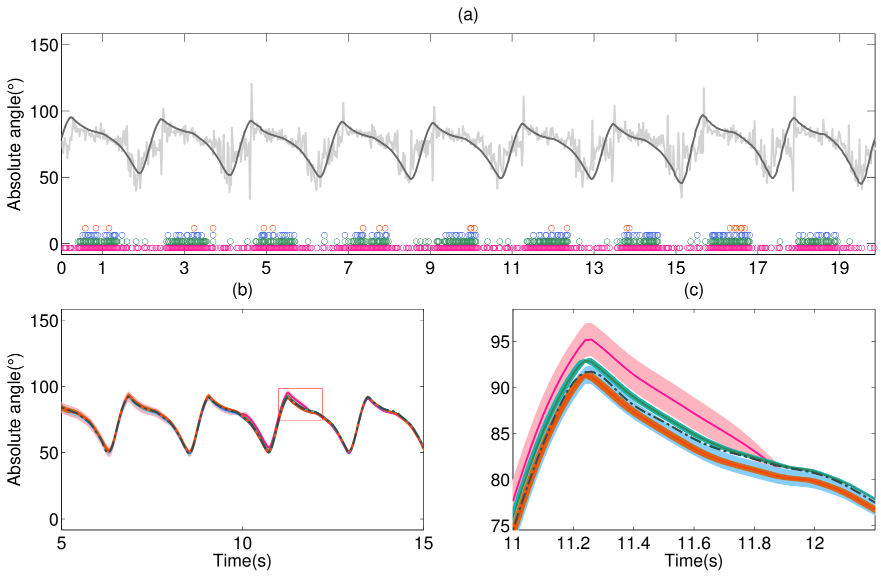

Figure 3: **Absolute shank angle of the Matricial Local Model**

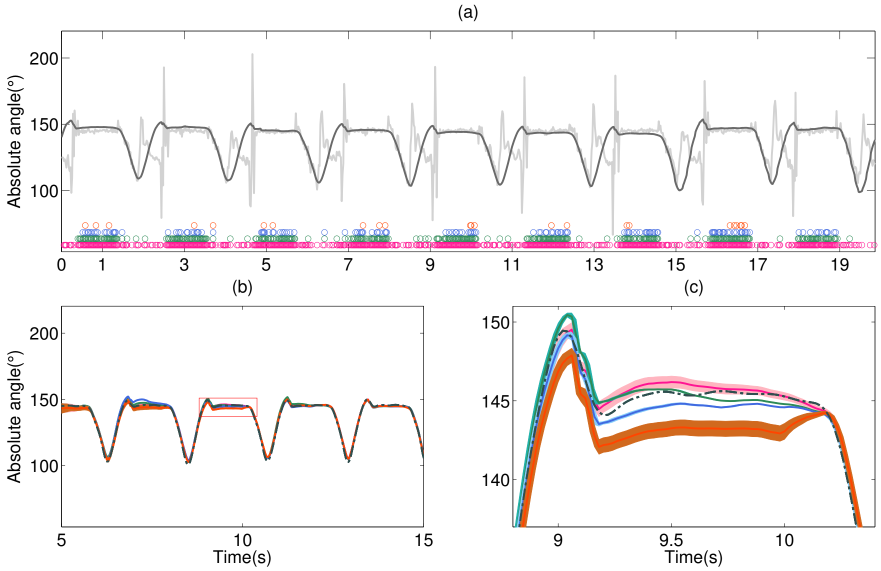

Figure 4: **Absolute foot angle of the Matricial Local Model**

2 Matricial Global KF results

In this section are shown the results for the matricial global KF.

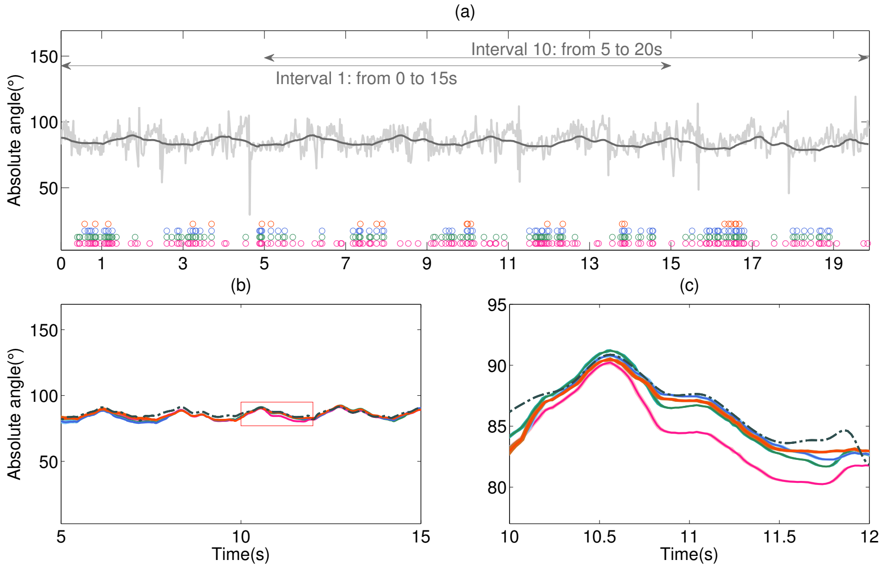

Figure 5: **Absolute trunk angle of the Matricial Global Model**. Where in (a) the gray and dark gray signals are respectively the accelerometer and gyroscope data. The circles are instants fulfilling the criterion Ψ with matrix approach, where the colors orange, blue, green and pink represent Ψ being defined respectively by equations (15) to (18) from the paper. The sub-figures (b) and (c) are the absolute trunk angles estimation.

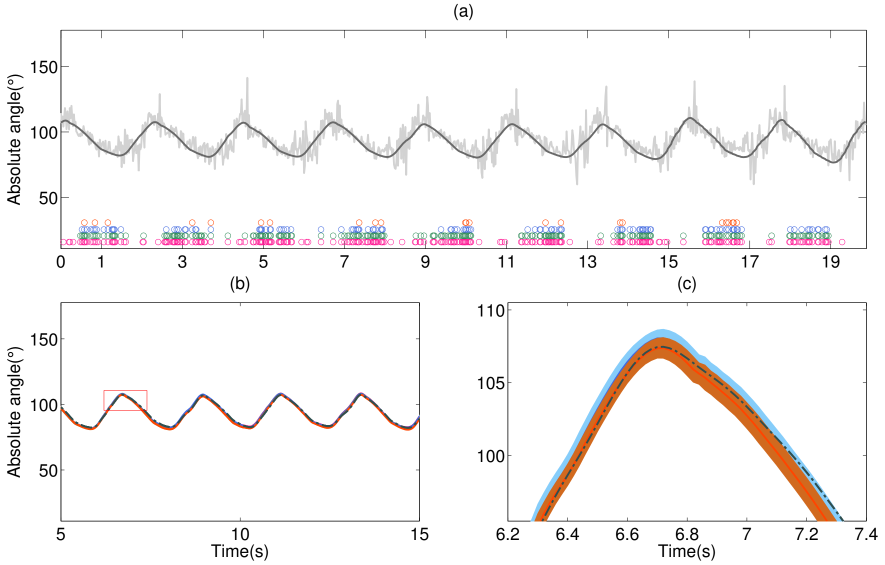

Figure 6: **Absolute thigh angle of the Matricial Global Model**

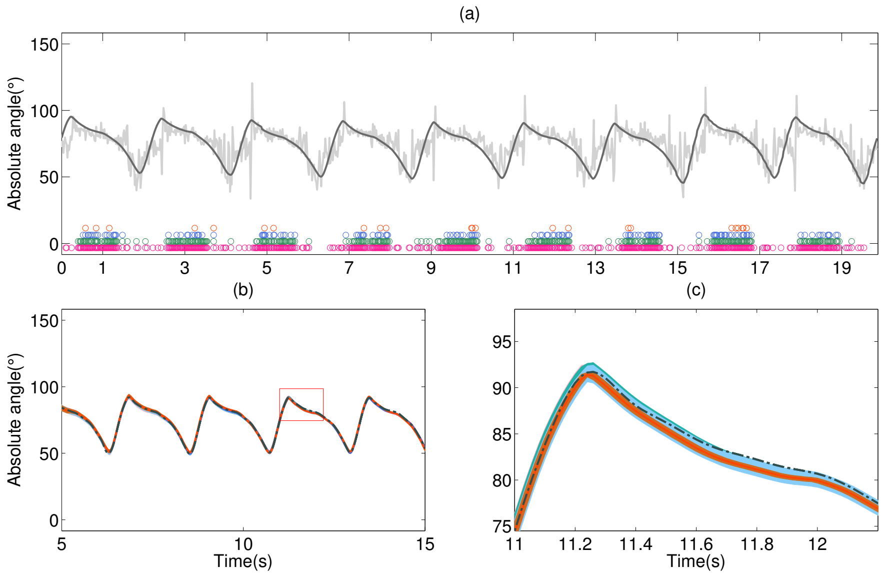

Figure 7: **Absolute shank angle of the Matricial Global Model**

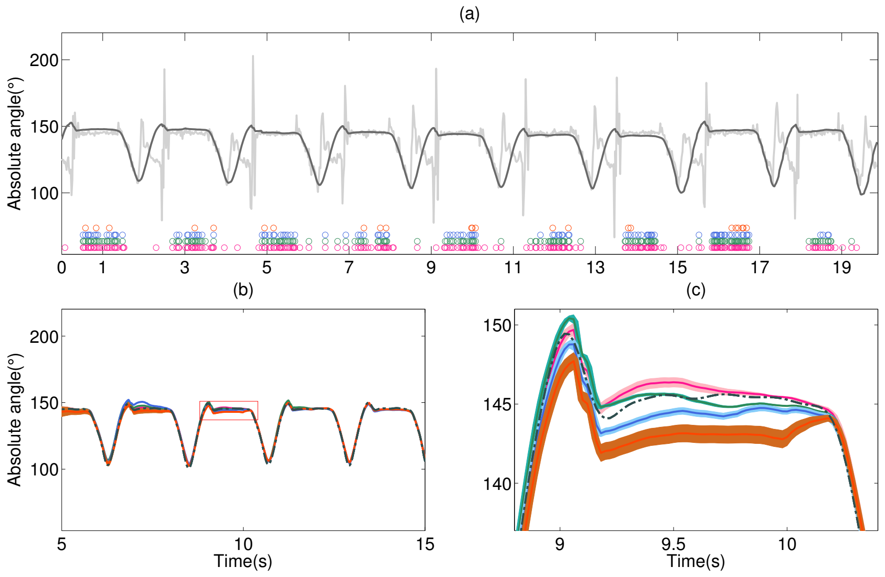

Figure 8: **Absolute foot angle of the Matricial Global Model**

3 Local vs Global filters

In this section we contrast the matricial local filter to the global filters (matricial global and MJLS).

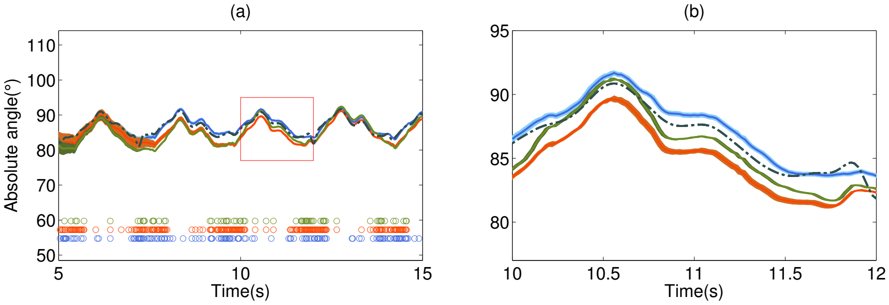

Figure 9: **Absolute trunk angle with all filters.** Where the colors olive, orange and blue represent respectively the approaches: matrix global, matrix local both with $\gamma = 2$ and Markovian local. The circles are the instants in which the criteria Ψ were satisfied.

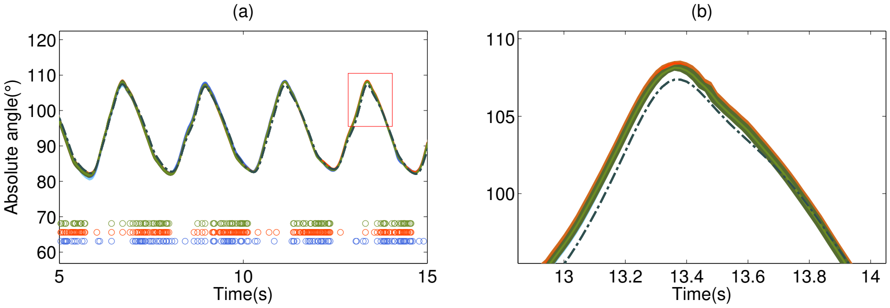

Figure 10: **Absolute thigh angle with all filters.**

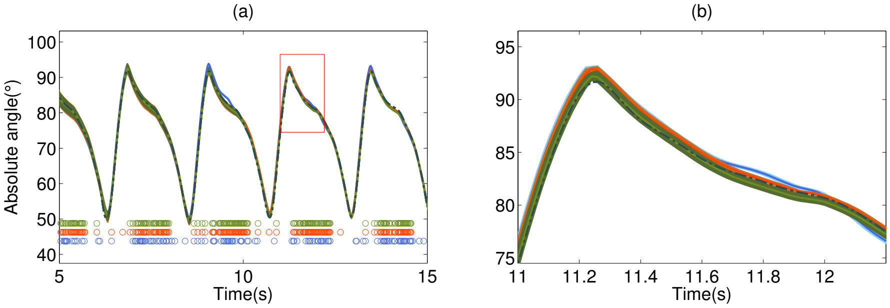

Figure 11: **Absolute shank angle with all filters.**

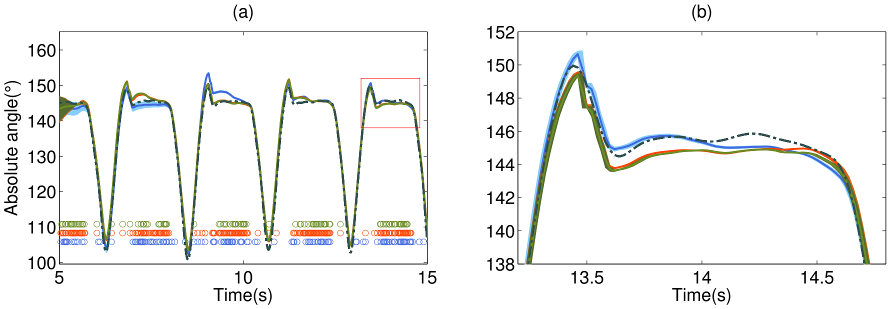

Figure 12: **Absolute foot angle with all filters.**
